# The Anti-Inflammatory Role of Vitamin E in Prevention of Osteoporosis

**DOI:** 10.1155/2012/142702

**Published:** 2011-11-17

**Authors:** A. S. Nazrun, M. Norazlina, M. Norliza, S. Ima Nirwana

**Affiliations:** Department of Pharmacology, Faculty of Medicine, The National University of Malaysia, 50300 Kuala Lumpur, Malaysia

## Abstract

There is growing evidence that inflammation may be one of the causal factors of osteoporosis. Several cytokines such as IL-1, IL-6, RANKL, OPG, and M-CSF were implicated in the pathogenesis of osteoporosis. These cytokines are important determinants of osteoclast differentiation and its bone resorptive activity. Anticytokine therapy using cytokine antagonists such as IL-receptor antagonist and TNF-binding protein was able to suppress the activity of the respective cytokines and prevent bone loss. Several animal studies have shown that vitamin E in the forms of palm-derived tocotrienol and *α*-tocopherol may prevent osteoporosis in rat models by suppressing IL-1 and IL-6. Free radicals are known to activate transcription factor NF*κ*B which leads to the production of bone resorbing cytokines. Vitamin E, a potent antioxidant, may be able to neutralise free radicals before they could activate NF*κ*B, therefore suppressing cytokine production and osteoporosis. Vitamin E has also been shown to inhibit COX-2, the enzyme involved in inflammatory reactions. Of the two types of vitamin E studied, tocotrienol seemed to be better than tocopherol in terms of its ability to suppress bone-resorbing cytokines.

## 1. Introduction

Osteoporosis is a bone disease, characterized by low bone mass and increased risk of fractures [1]. It is well accepted that osteoporosis can be caused by various endocrine, metabolic, and mechanical factors. However, recently, there are opinions that there may be an inflammatory component in the etiology of osteoporosis [2, 3]. There is plenty of evidence linking inflammation to osteoporosis. Epidemiological studies have identified higher incidence of osteoporosis in various inflammatory conditions such as ankylosing spondylitis, rheumatoid arthritis, and systemic lupus erythematosus [4–7]. This association was also observed clinically whereby the degree of osteoporosis was equivalent to the extent of inflammation. If the inflammation was systemic, bone loss will occur at all skeletal sites, whereas if the inflammation was only restricted to a site, bone loss will only occur locally at that site of inflammation [3]. Elderly patients are more prone to osteoporosis, and this was believed to be connected to the elevated production of proinflammatory cytokines with aging [8, 9]. 

The occurrence of inflammation is indicated by the presence of inflammatory markers such as cytokines and C-reactive protein. Biochemical studies have demonstrated elevation of proinflammatory cytokines TNF-*α* and IL-6 in arthritic disease such as gouty arthritis, rheumatoid arthritis, and psoriatic arthritis [10, 11]. An obvious relationship between inflammation and osteoporosis was seen in rheumatoid arthritis, whereby proinflammatory cytokines were released causing bone loss around the affected joints [12]. The level of C-reactive protein, a sensitive marker of systemic inflammation, was also found to be associated with bone mineral density [13]. Inflammation may contribute to bone loss by affecting the bone remodeling process, favouring bone resorption activity by osteoclasts rather than bone formation activity by osteoblasts [14, 15]. Bone resorption is determined by the balance between two cytokines, receptor activator of nuclear factor *κ*B ligand (RANKL), and osteoprotegerin (OPG) [16]. RANKL is crucial for the differentiation and activation of osteoclast [17]. Higher RANKL levels were associated with lower bone mineral density in men [18]. Administration of serum RANKL to mice promoted osteoclast growth and activation, leading to osteoporosis [19]. On the other hand, OPG antagonizes RANKL by binding with RANKL and preventing it from binding to RANK receptors. By doing that, OPG was able to inhibit osteoclastogenesis and bone resorption [20]. Macrophage colony stimulating factor (MCSF) is another important determinant of osteoclastogenesis, but its mechanism to modulate osteoclastogenesis is still not clear [20].

The “upstream” cytokines such as IL-1, IL-6, and TNF-*α* [21, 22] and “downstream” cytokines such as RANKL, OPG, and M-CSF [23–25] played an important role in bone remodeling. Imbalance in their bioactivity may lead to bone loss and osteoporosis. Cytokines are small- to medium-sized proteins or glycoproteins with molecular weight ranging from 8 to 40,000 dalton. They act as the biological mediator for most cells and function at low concentrations between 10^−10^ and 10^−5^ molar. They have a short half-life of less than 10 minutes, and their serum level can be as low as 10 pg/mL. The cytokine levels increase dramatically during inflammation and infection. The measurement of cytokine levels in close vicinity to bone such as the bone marrow is important for studies on osteoporosis and other bone diseases. In postmenopausal women, cytokine production by the peripheral monocytes correlated well with cytokines secreted by monocytes in the bone marrow. Therefore, cytokine levels in the serum are representative of the local monocytes [26]. Stromal cells and osteoblasts produce interleukin-1, interleukin-6, and tumor necrosis factor-*α*. These proinflammatory cytokines are also known as the bone-resorbing cytokines or proosteoclast cytokines as they promote osteoclast differentiation and activity [27–30]. The bone resorption activity of these cytokines in ovariectomised rats was reduced with anticytokine therapy such as IL-1 receptor antagonists and TNF-binding protein [31]. Vitamin E, a potent antioxidant vitamin, was also found to inhibit or suppress cytokine production [32, 33]. This vitamin E action may be responsible for its ability to prevent inflammation and osteoporosis, seen in several studies on osteoporosis using animal models [34].

Vitamin E is a group of potent, lipid-soluble, chain-breaking antioxidants. It can be classified into tocopherol and tocotrienol based on the chemical structure. Palm oil, which is extracted from the pulp of the fruit of the oil palm *Elaeis guineensis*, is abundant in tocotrienols. Tocotrienol has an unsaturated farnesyl (isoprenoid) side-chain, while tocopherol has a saturated phytyl side chain [35]. 

Vitamin E occurs in eight isoforms of *α*-, *β*-, *γ*-, and *δ*-tocopherols or tocotrienols. It was thought that both the *γ* and *δ* isomers of tocopherol have better antioxidant and anti-inflammatory activities than the *α* isomer [36, 37]. Once vitamin E is absorbed in the intestine, it will enter the circulation via the lymphatic system and be transported to the liver with the chylomicrons [38]. Vitamin E is metabolized by cytochrome P450 and then excreted in the urine [39]. 

In human subjects and animal models, high doses of vitamin E were found to exhibit anti-inflammatory effects by decreasing C-reactive protein (CRP) and inhibiting the release of proinflammatory cytokines [40]. These were evident in a study on patients with coronary artery disease, whereby the CRP and tumor necrosis factor-*α* (TNF-*α*) concentrations were found to be significantly lowered with *α*-tocopherol supplementation compared to placebo [41]. Since vitamin E was also found to inhibit cyclooxygenase-2 activities, it was thought to be able to exert anti-inflammatory and anticarcinogenic activities, especially in the colon [42]. This was demonstrated by Yang et al. [43], who found that vitamin E was able to significantly lower colon inflammation index and reduced the number of colon adenomas in mice given azoxymethane. 

This paper will focus on the effects of vitamin E on bone-resorbing cytokines with special attention on IL-1 and IL-6.

## 2. Interleukin-1 (IL-1)

IL-1 plays an important role in various reactions towards infection, inflammation, and immune activation. This cytokine is produced by various cells but the main producer is the monocyte. In the physiological condition, monocytes do not secrete IL-1 but, under pathological conditions such as septic shock, IL-1 is rapidly released and acts directly on the blood vessels. Other cytokines such as TNF-*α* and interferon, bacterial endotoxin, virus, and antigen can also stimulate the release of IL-1. Reactive oxygen species such as superoxide radicals have been shown to induce IL-1 production [32, 44]. IL-1 is involved in the pathogenesis of various diseases associated with bone loss such as osteoporosis [45, 46], cancer-induced osteolysis [47], rheumatoid arthritis [48], and osteolysis of orthopedic implants [49]. IL-1 is also an important factor in both *in vivo* and *in vitro* bone resorption [50, 51]. It stimulates the formation and activity of osteoclasts, leading to excessive bone resorption. Suda et al. [52] demonstrated that the presence of osteoblast and stromal cells was crucial in the formation of osteoclasts by IL-1. Thomson et al. [53] also reported that osteoblasts secrete a factor that stimulates the bone-resorbing activities of rat osteoclasts. However, Xu et al. [54] demonstrated that rat osteoclasts expressed mRNA to IL-1 receptors, while Yu and Ferrier [55] found that osteoclast is one of the target cells for IL-1. These studies proved that IL-1 can act directly on osteoclasts without the presence of osteoblasts or stromal cells. IL-1 may also promote formation of osteoclasts [56]. It acts by activating nuclear factor *κ*B (NF*κ*B) in osteoclast and prevents its apoptosis [57]. It was found that the estrogen-deficient state in postmenopausal women or ovariectomised rats resulted in increased production of IL-1 by monocyte and other bone marrow cells [58, 59]. Estrogen replacement or IL-receptor antagonist was able to prevent the elevation of IL-1 in ovariectomised rats [60, 61]. Vitamin E was also found to have the ability to suppress IL-1 production by activated monocytes [62]. In a different study, combination of superoxide dismutase and vitamin E was effective in inhibiting IL-1 production by human monocytes [32]. The ability of vitamin E to inhibit IL-1 in the bone environment may have prevented bone loss. 

## 3. Interleukin-6 (IL-6)

IL-6 is another cytokine that is associated with various pathophysiological processes in humans. It is produced by the haematopoetic and nonhaematopoetic cells when they were exposed to various types of stimulation. During bone remodeling, IL-6 is produced in nanomolar concentrations by stromal cells and osteoblasts under the influence of parathyroid hormone, vitamin D_3_, growth factor, and other cytokines [63]. IL-6 was also reported to be produced by osteoblasts when stimulated by IL-1, TNF-*α*, and lipopolysaccharide [64]. McSheeny and Chambers [65] reported that osteoblasts were stimulated by local IL-1 to produce IL-6, which was responsible for the activation of osteoclasts. IL-6 promoted the differentiation of osteoclasts from its precursor and played an important role in the pathogenesis of osteoporosis due to estrogen deficiency [66, 67]. The IL-6 elevation in postmenopausal women was reduced by estrogen replacement therapy [68]. The elevation of IL-6 may be related to free radical activities especially reactive oxygen species. Reactive oxygen species was found to elevate the IL-6 levels directly via activation of nuclear factor *κ*B (NF*κ*B) [69]. High cytokine levels would also result in activation of NF*κ*B and promotion of osteoclastogenesis [70]. 

## 4. Vitamin E as Anticytokine Agent

The effects of vitamin E on bone resorbing cytokines for prevention and treatment of osteoporosis have been studied using FeNTA and nicotine rat models [34, 71]. These models represent osteoporosis caused by oxidative stress and smoking, respectively. However, similar studies in humans are still lacking. Ferric nitrilotriacetate (FeNTA) is an oxidizing agent which produces free radicals via the Fenton reaction [72, 73]. Oxidative stress can be induced in rats by injecting them with FeNTA, allowing the hazardous effects of free radicals on various organs and tissues including bone to be studied. The bone resorbing cytokines, IL-1 and IL-6, were found to be elevated in this oxidative stress rat model, indicating inflammation. This was accompanied by osteoporotic changes as indicated by the measurement of bone markers and histomorphometric parameters [34]. The elevation of cytokines was probably achieved through the activation of cytokine-encoding genes like STAT3 or nuclear factor-kappaB by the free radicals [74, 75]. Therefore, there exist relationships between free radicals, inflammation, and bone loss which can lead to osteoporosis. When vitamin E in the form of tocotrienols and *α*-tocopherol were supplemented to these rats, IL-1 and IL-6 elevations were suppressed. Concurrent with this, the osteoporotic changes were also inhibited [34, 71, 76]. Therefore, there is a possibility that vitamin E, a potent antioxidant, has prevented free radicals from causing inflammation and osteoporosis. Tocotrienols seemed to be more superior than *α*-tocopherol in suppressing proinflammatory cytokines in the FeNTA rat model and in protecting their bone against osteoporosis [34]. Both the tocopherol and tocotrienol may have achieved this by scavenging the free radicals generated by FeNTA before they could activate the monocytes and osteoblasts, cells that produce IL-1 and IL-6.

 Cigarette smoking is a modest risk factor for osteoporosis [77]. Nicotine is among the 4,700 chemicals found in the tar phase of cigarette smoke [78]. Nicotine injected into rats can be used as a model for osteoporosis related to smoking. Various animal studies have confirmed the deleterious effects of nicotine on bone remodeling [79–85]. Nicotine inhibited osteoblast activity and growth [86, 87] but stimulated osteoclast activity [83]. Nicotine has also been shown to induce oxidative stress in both *in vitro *and *in vivo* animal studies [88, 89]. Crowley-Weber et al. [90] had reported that other than oxidative stress, nicotine also activated nuclear transcription factor-*κ*B (NF-*κ*B) in the tissues of smokers. The activation of NF-*κ*B-signaling pathway may be the mechanism for bone loss as it is responsible for osteoclast differentiation [76, 91]. Nicotine has been shown to significantly elevate the proinflammatory cytokines IL-1 and IL-6 in rats. Using the same model, tocotrienol was able to prevent nicotine-induced elevation of IL-1 and IL-6, while tocopherol had no significant effects on both cytokines [71]. Tocotrienol was more effective compared to tocopherol in terms of its action on bone resorbing cytokines and therefore was more effective in reducing inflammation and bone loss. 

## 5. Anti-Inflammatory Action of Vitamin E in Prevention of Osteoporosis 

Results from studies on cytokines have given us some insight on the mechanisms involved in the protection of vitamin E against osteoporosis. Free radicals are known to activate transcription factor NF*κ*B which leads to the production of bone resorbing cytokines interleukin-1 and interleukin-6. These proinflammatory cytokines were believed to provide the link between inflammation and osteoporosis. Vitamin E may scavenge and neutralize free radicals before they could activate transcription factor NF*κ*B. This was seen in an oxidative stress model (FeNTA model) in which vitamin E had reduced the levels of bone-resorbing cytokines [34]. Alternatively, vitamin E may have prevented the activation of NF*κ*B by enhancing the internal antioxidative enzymes within the bone. This was demonstrated by Maniam et al. [92], whereby vitamin E supplementation reduced the femoral thiobarbituric acid-reactive substance (TBARS) and increased the glutathione peroxidase activity.

Since osteoporosis is associated with inflammation, there is also a possibility that Vitamin E may have some anti-inflammatory action. Yam et al. [93] found that tocotrienol was able to suppress cyclooxygenase-2 (COX-2) expression in RAW 264.7 cells that were exposed to lipopolysaccharide. COX-2 is an inducible enzyme expressed during inflammation. A RAW cell is a macrophage-like cell which transformed into preosteoclasts when RANKL is added. This suggested that vitamin E may act as anti-inflammatory agent in protecting bone against excessive osteoclastic activity. Previous study has shown that aspirin or other nonsteroidal anti-inflammatory drugs (NSAID) inhibited NF*κ*B [94]. Similar to tocotrienol, these anti-inflammatory drugs inhibit COX-2. As the activation of NF*κ*B is linked to proinflammatory cytokines and inflammation, it further provides evidence of the anti-inflammatory role of tocotrienol in preventing osteoporosis.

Based on the results from the studies above, tocotrienol was more superior than tocopherol in terms of its ability to suppress bone resorbing cytokines. The more superior tocotrienol action may be contributed by its more potent antioxidant property. It has better interaction with lipoprotein in membrane lipids and is uniformly distributed in the membrane layer compared to tocopherol [35, 95]. Tocotrienol was also better at maintaining the antioxidant status within the rat bone compared to tocopherol [92]. Thus, the antiosteoporotic effect of tocotrienol may be partly explained by its anti-inflammatory as well as antioxidative effects.
